# Non-invasive Biomarkers of Acute Rejection in Kidney Transplantation: Novel Targets and Strategies

**DOI:** 10.3389/fmed.2018.00358

**Published:** 2019-01-08

**Authors:** Michael Eikmans, Els M. Gielis, Kristien J. Ledeganck, Jianxin Yang, Daniel Abramowicz, Frans F. J. Claas

**Affiliations:** ^1^Department of Immunohematology and Blood Transfusion, Leiden University Medical Center, Leiden, Netherlands; ^2^Laboratory of Experimental Medicine and Pediatrics, University of Antwerp, Antwerp, Belgium; ^3^Department of Nephrology and Hypertension, Antwerp University Hospital, Antwerp, Belgium

**Keywords:** kidney transplantation, acute rejection, transplant outcome, biomarker, non-invasive, transcriptomics, proteomics, cell-free DNA

## Abstract

Kidney transplantation is considered the favored treatment for patients suffering from end-stage renal disease, since successful transplantation is associated with longer survival and improved quality of life compared to dialysis. Alloreactive immune responses against the donor kidney may lead to acute rejection of the transplant. The current diagnosis of renal allograft rejection mainly relies on clinical monitoring, including serum creatinine, proteinuria, and confirmation by histopathologic assessment in the kidney transplant biopsy. These parameters have their limitations. Identification and validation of biomarkers, which correlate with or predict the presence of acute rejection, and which could improve therapeutic decision making, are priorities for the transplantation community. There is a need for alternative, less invasive but sensitive markers to diagnose acute graft rejection. Here, we provide an overview of the current status on research of biomarkers of acute kidney transplant rejection in blood and urine. We specifically discuss relatively novel research strategies in biomarker research, including transcriptomics and proteomics, and elaborate on donor-derived cell-free DNA as a potential biomarker.

## Features Of Acute Kidney Transplant Rejection

### Kidney Transplantation

**Figure 1:**
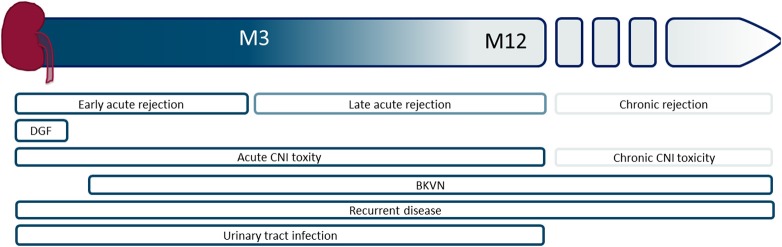
Schematic overview of the timing of different graft-associated complications, starting from the time of transplantation to the moment of graft failure. M, month; DGF, delayed graft function, CNI, calcineurin inhibitor; BKVN, BK virus nephropathy.

Kidney transplantation is the treatment of choice for patients with end-stage renal failure. Patient survival and quality of life after transplantation are superior compared to remaining on dialysis (1, 2). The transplanted organ is predisposed to a number of acute insults related to immunologic injury, ischemia–reperfusion injury (IRI), medication toxicity, and surgical complications (3, 4). Figure 1 represents an overview of complications that can be encountered.

Introduction of more potent immunosuppressive drugs has led to a diminished incidence of acute rejection. Still, 10% of the kidney transplant recipients develop an acute rejection within the first year (5). Acute rejection is generally well-treatable by intravenous steroids and/or anti-thymocyte globulin. But once it occurs, it may have an adverse impact on graft outcome (6).

### Acute Rejection Types and Underlying Pathophysiologic Mechanisms

Acute rejection episodes are most prevalent in the first weeks after transplantation (7), and can be categorized into T cell-mediated (TCMR) and antibody-mediated (ABMR) rejection. In TCMR, lymphocytes infiltrate and proliferate within the interstitial space. These lymphocytes may induce cytotoxic effects on the renal tubular epithelial cells, thereby causing tubulitis. Vascular rejection is identified when mononuclear cells invade arteries thereby causing arteritis and eventually severe transmural necrosis of blood vessels.

**Figure 2:**
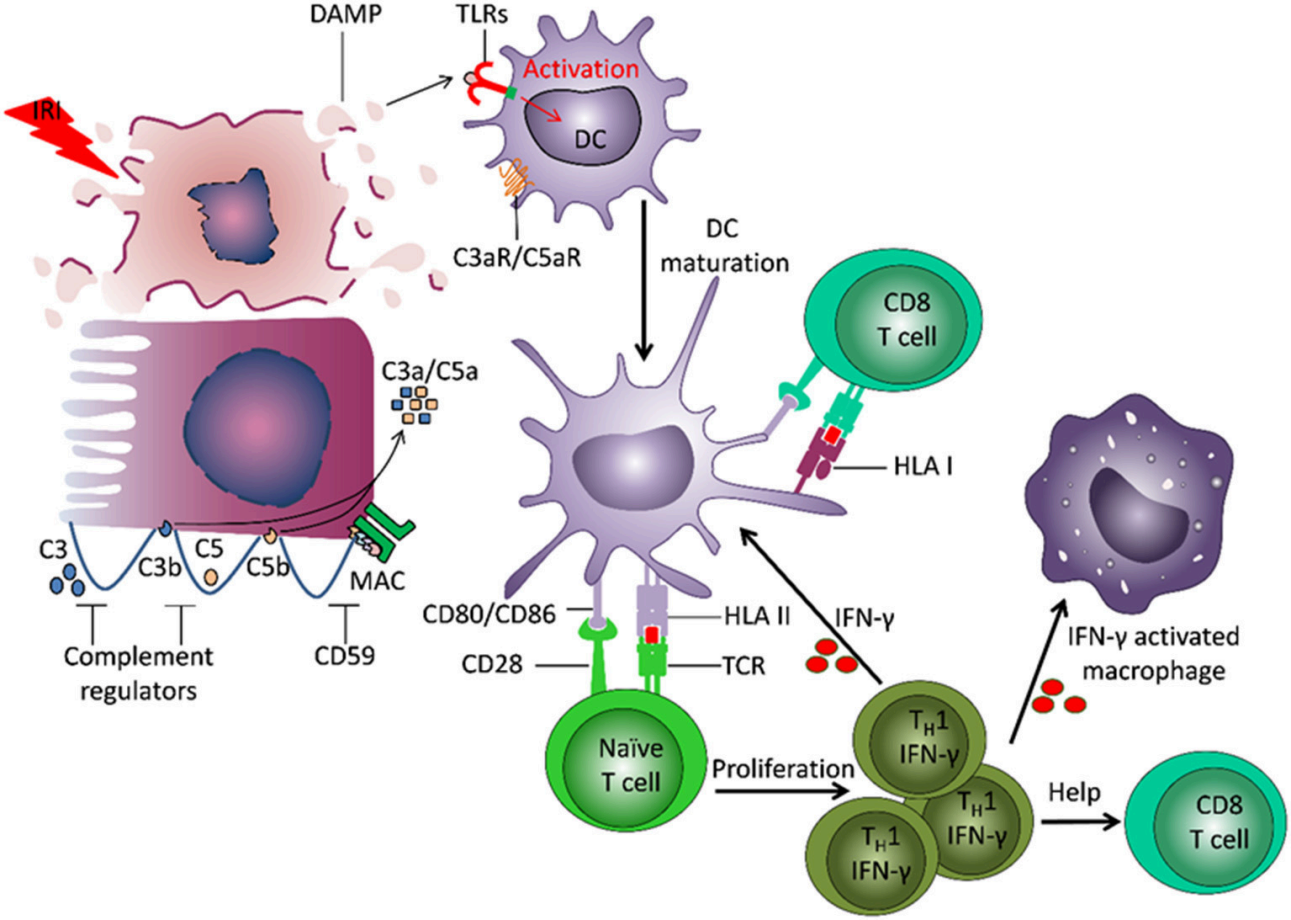
Involvement of innate and adaptive immunity in development of damage to the transplant. Ischemia reperfusion injury (IRI) leads to induction of necrosis of tubular cells and release of damage associated molecular patterns (DAMPs), which are normally hidden within intact cells. DAMPs bind to Toll-like receptors (TLRs) on dendritic cells (DC) and induce their activation and maturation. The matured DC present donor-derived HLA antigens and co-stimulatory molecules to naïve T cells, which drives T cell differentiation into IFNγ producing TH1 cells. IFNγ can stimulate maturation of other DCs, induce macrophage activation and recruitment, and direct differentiation of CD8^+^ T cells. The recipient DCs are also able to capture and present donor HLA antigens, and thereby stimulate recipient CD8^+^ T cells. IRI can lead to induction of a local increase of complement component 3 (C3). Cleavage of C3 by the alterative pathway results in C3b deposition on the cell membrane and complement cascade activation. The small fragments C3a and C5a, released during complement activation, have pro-inflammatory effects. The formation of membrane attack complex (MAC) leads to target cell lysis and release of DAMPs.

The adaptive immune system plays a central role in TCMR (Figure 2). The frequency of alloreactive T cells (naïve, memory) is 1–10% (8). Direct allorecognition is mediated by interaction between the T cell receptor (TCR) on recipient T cells and mismatched human leukocyte antigens (HLA) on donor derived antigen-presenting cells (APCs). Indirect allorecognition also plays a role: donor-derived HLA antigens are processed and presented by recipient APCs to CD4^+^ T cells (9). HLA/peptide-TCR interaction and co-stimulation signals promote T cell proliferation and differentiation. CD8+ T cells release perforin and granzyme B that induce apoptosis of target cells (10). Monocytes and myeloid DCs also infiltrate the graft and contribute to acute rejection (11, 12).

ABMR, mediated by donor specific antibodies (DSA) that target HLA- or non-HLA antigens on the donor endothelium, can be seen within the first year post-transplant (13, 14). Antigen-antibody interaction results in antibody-dependent cellular cytotoxicity and complement activation, causing lysis of target cells. Endothelial cell injury leads to platelet aggregation and recruitment of leukocytes via cytokines, chemokines, and chemoattractants. These phenomenons may lead to graft failure (15).

### Cell Death in the Kidney and Innate Immunity

The innate immune system offers non-specific defense against invading pathogens: it prevents infection via the complement system and cellular responses by macrophages and DCs. These cells carry Toll-like receptors (TLRs), which recognize pathogen-associated molecular patterns on pathogens.

Innate immunity contributes to transplant injury (Figure 2). IRI leads to necrosis of tubular cells and release of damage associated molecular patterns (DAMPs), molecules hidden within intact cells but released from damaged cells (16, 17). DAMPs bind to TLRs on DCs and induce their activation and maturation. Apoptosis of tubular cells contributes to transplant rejection (18, 19). DCs present donor-derived HLA antigens and co-stimulatory molecules to naïve T cells, driving their differentiation into IFNγ-producing Th1 cells. IFNγ can induce differentiation and activation of myeloid cells and CD8^+^ T cells. IRI can also lead to local activation of the complement cascade.

### Monitoring the Transplant: Conventional Parameters

Monitoring of kidney transplant patients is an important part of post-transplantation management (20). According to KDIGO (Kidney Disease Improving Global Outcomes) guidelines (https://kdigo.org/) acute kidney injury is defined as a rise in serum creatinine levels >0.3 mg/dL in 48 h, a percentage increase in serum creatinine of ≥50% (1.5-fold from baseline), or a reduction in urine output (oliguria <0.5 mL/kg/h for more than 6 h). It is recommended to quantify protein excretion every 3 months during the first year. Polyoma virus and Epstein-Barr virus screening using plasma nuclear acid testing needs to be performed monthly post-transplant during the first 3 months and every 3 months thereafter until 1 year.

The performance of a renal needle biopsy is necessary in case of an unexplained increase in serum creatinine. The Banff classification of allograft rejection provides standardized criteria for the histologic diagnosis of acute rejection, thereby scoring inflammation in the different renal compartments (21). Lesions in the microcirculation, together with C4d deposition in peritubular capillaries and donor-specific antibodies in the patient's serum, point to ABMR.

Routine immunologic laboratory tests are applied for determining immunologic sensitization of the patient and for assessing risk of adverse graft outcome. The complement-dependent cytotoxicity test is performed before transplantation, and its introduction has led to significant reduction in the incidence of hyperacute rejection (22). Likewise, pre-transplant HLA alloantibody screening helps in optimizing donor selection. Post-transplant HLA alloantibody screening is used to define the type of acute rejection, and it aids in establishing the possible impact of antibodies on graft function.

## Non-Invasive Biomarkers For Acute Kidney Transplant Rejection

### Limitations of Conventional Parameters

Alterations in serum creatinine are not specific for graft injury: a change in the level may indicate an intrinsic renal process, such as acute rejection, graft infection; or a transient process, such as hemodynamic effects of calcineurin inhibitors or pre-renal volume depletion (20). Furthermore, the process of acute rejection involves different stages, with clinical signs of graft damage occurring late in the continuum, after a stage of subclinical graft damage (15, 23). In other words, serum creatinine may not change despite considerable injury to the kidney. The performance of a biopsy may lead to complications for the transplant recipient (24). Since hospitalization is required for all biopsy procedures, it represents an expensive examination. Other limitations of a renal biopsy include sampling error and interobserver variability between pathologists (25).

Altogether, there is a need for alternative, less invasive but sensitive markers to diagnose acute graft rejection. Identification and validation of biomarkers, which correlate with and/or predict the presence of acute rejection, and which could improve therapeutic decision making, are priorities for the transplantation community (26). By serial sampling, development of rejection might be anticipated before the actual development of tissue injury. By being able to distinguish high-risk from low-risk patients according to biomarker information, individualization of immunosuppressive drug therapy may be facilitated.

### Requirements for a Biomarker

According to the National Institutes of Health (NIH) Biomarker Definition Working Group a biomarker is defined as “a characteristic that is objectively measured and evaluated as an indicator of normal biological processes, pathogenic responses, or pharmacological responses to a therapeutic intervention.” Gwinner has stated criteria of a biomarker in disease (27). In kidney transplantation an ideal biomarker should rapidly, accurately, inexpensively and non-invasively identify subjects with incipient allograft injury, and discern the type of injury. Clinically useful assays have a high sensitivity, specificity, and negative and positive predictive values (NPV, PPV), and a diagnostic area under the receiver curve (AUC) nearing 1.0 (26).

Research groups have studied potential biomarkers to monitor different disease entities after kidney transplantation. Besides distinguishing occurrence of acute rejection or development of chronic damage (fibrosis), biomarkers may be applied to assess the effect of an intervention trial after transplantation or to estimate the extent of immunological tolerance that a transplant recipient has developed toward its donor graft. Studies that have investigated potential biomarkers for renal acute rejection will be discussed in the subsequent paragraphs, and are summarized in Table 1.

**Table 1:** Overview of the diagnostic performance of biomarkers in detecting kidney transplant rejection.

**Authors**	**Biomarkers**	**Sample size**	**Rejection type**	**AUC**	**Sensitivity%**	**Specificity%**	**PPV%**	**NPV%**
Suthanthiran et al. (28)	Three-gene signature in urine cell pellets	*N* = 485 kidney transplant patients *N* = 4,300 urine samples	Acute TCMR	0.74	71	72		
Roedder et al. (29)	kSORT	*N* = 436 kidney transplant patients *N* = 558 blood samples	Acute rejection (both TCMR and ABMR)	0.94	83.0	90.6	93.2	
Hricik et al. (30)	CXCL9 protein	*N* = 255 kidney transplant patients	Banff ≥ 1 rejection	0.86	85.2	80.7	67.6	92
Rabant et al. (31)	CXCL9	*N* = 247 kidney transplant patients *N* = 290 matched kidney biopsies and urine samples	TCMR	0.86	80	87	23.5	98.9
Rabant et al. (31)	CXCL10	*N* = 247 kidney transplant patients *N* = 290 matched kidney biopsies and urine samples	ABMR	0.70	73	61.6	25.7	92.6
Rabant et al. (31)	CXCL10	*N* = 247 kidney transplant patients *N* = 290 matched kidney biopsies and urine samples	Mixed rejections	0.80	74.2	83.3	40.4	95.5
Bloom et al. (32)	dd-cfDNA	*N* = 102 kidney transplant patients *N* = 107 plasma samples matched with a biopsy.	Active rejection	0.74	59	85	61	84

*AUC, area under the curve; NPV, negative predictive value; PPV, positive predictive value; CXCL, chemokine (C-X-C motif) ligand; ABMR, antibody mediated rejection; TCMR, T cell mediated rejection; dd-cfDNA, donor-derived cell free DNA*.

### Biomarkers of Acute Rejection: Where Are We Now?

Biomarkers are often investigated in a biased approach, by looking at molecules that are expected to play a role in the pathophysiology of rejection. Research groups have investigated general immune cell subsets (T cells, B cells, monocytes), but also immune activation through chemokines CXCL9 and CXCL10, the immune effector pathway of cytotoxic T cells (granzyme B, perforin, FasL), and donor-specific reactivity according to the number of T cells that produce IFNγ in reaction to allogeneic donor cells (33). Molecules that more generally reflect injury to the renal parenchyma including kidney injury molecule 1, neutrophil gelatinase-associated lipocalin, and cell-free (cf)DNA have also frequently been studied. Several of the molecules mentioned will be discussed further in the next sections.

With unbiased approaches in biomarker research, a large number of molecules are investigated at the same time, in a non-hypothesis-based manner. The development of omics technologies, including transcriptomics, proteomics, and metabolomics, which quantify the abundance of gene transcripts (mRNA), proteins, and metabolites, respectively, in cell/tissue extracts or biofluids has opened up new opportunities in the non-invasive diagnosis of acute rejection (34).

### Transcriptomics

Over the last years, the group from Halloran has performed molecular analyses of transplant biopsies to enrich the standard pathology approach by this so-called molecular microscope strategy (35, 36). Other groups have analyzed molecular markers in urine and blood.

Suthanthiran's group analyzed mRNA transcripts in urine sediments of renal transplant recipients, including CD3ε, perforin, granzyme B, proteinase-inhibitor-9 (inhibitor of granzyme B), CD103 (intraepithelial homing of lymphocytes), CXCL10, and CXCR3 (chemokine receptor on lymphocytes). A three-gene signature for the diagnosis of acute rejection was identified in both a discovery and validation cohort (28). Interestingly, the signature was increased up to 20 days before histological diagnosis by biopsy.

Sarwal's group introduced the kSORT (kidney solid organ response test) comprising peripheral blood transcriptome assessment. Within a multicenter study, 17 genes distinguished acute rejection from no-rejection (29). The kSORT gene signature was designed based on pathway and network analysis. Ten genes were previously put forth as peripheral biomarkers for acute rejection and the others were expressed in activated monocytes, endothelial cells, and T cells. The 17-gene model allowed for the determination of a probability score for acute rejection (0–100%). This model was validated in an independent cohort of patients, and within a prospective cohort it was shown that 84% of the samples at an episode of acute rejection were correctly classified. Acute rejection could be predicted already 3 months before histological diagnosis in 64% of cases at time of stable graft function. An algorithm was developed and commercialized to report a risk score for acute rejection (high–intermediate–low risk), thereby reflecting the patient's immune response at the time of assessment.

### Proteomics

The most promising and investigated protein biomarkers for acute renal rejection are the IFNγ-induced chemokines CXCL9 and CXCL10. These chemokines recruit T cells to inflammatory sites after binding with CXCR3 on activated T cells.

In a multicenter prospective study, Hricik and coworkers (30) observed higher urine levels of CXCL9 in patients with acute rejection (>Banff IA), already up to 30 days before the biopsy. Low urinary CXCL9 protein levels could be used to rule out acute rejection, as shown by a high NPV. Furthermore, low urinary CXCL9 protein levels at 6 months during stable graft conditions pointed to a low risk for the development of future acute rejection between 6 and 24 months (NPV 99.3%).

Rabant and colleagues showed that urinary CXCL9 was a strong predictor of TCMR (AUC 0.86), while CXCL10 showed a better performance to diagnose ABMR (AUC 0.70) and mixed rejections (AUC 0.80) compared to TCMR (31). Already at 1 month, during stable graft conditions, CXCL10 urine levels predicted subsequent acute rejection (AUC 0.72, NPV 93%) (37), suggesting that low chemokine levels predict immunological quiescence.

### Cell-Free DNA in Kidney Transplantation

Most DNA in the body is located within cells. However, a small amount of DNA in the form of cfDNA fragments circulates freely throughout the body. As the release of donor DNA in the recipient's blood is secondary to cell damage in the graft, these molecules may be biomarkers of allograft health. Lo et al were the first to report on the presence of donor-derived cell-free (dd-cf)DNA in plasma of transplant recipients (38).

A mean dd-cfDNA of 0.34% from 1 to 12 months post-transplant was observed in the plasma of stable kidney transplant recipients (39). Total cfDNA levels were increased during acute rejection and systemic infection and to a lesser extent during local infections, acute tubular necrosis, and drug-induced nephrotoxicity (40). Furthermore, in a small number of female recipients with male donor kidney grafts, increased dd-cfDNA levels were observed during acute rejection and graft infection episodes (40). In a small proof-of-principle study, increased dd-cfDNA levels were found during a rejection episode (41). In a cross-sectional study from Bloom et al. higher dd-cfDNA levels were found in plasma of recipients with an acute (TCMR with Banff ≥ IB and ABMR) or chronic active rejection compared to recipients without active rejection in their biopsy (32). The authors reported a threshold of 1% dd-cfDNA to distinguish rejection from no rejection (59% sensitivity, 85% specificity, PPV 61%, NPV 84%). The authors concluded that a plasma dd-cfDNA below 1% reflects the absence of an active rejection. It remains, however, remarkable that the dd-cfDNA fraction exceeded 1% in ~25% of the samples without active rejection. We recently demonstrated (42) that increases in dd-cfDNA above a reference baseline 0.88% were associated with acute rejection, but also with acute pyelonephritis and acute tubular necrosis. In this study, 18% of the increases in the dd-cfDNA fraction could be explained by the presence of one of these adverse events. Our data further demonstrate that plasma dd-cfDNA levels are not superior to serum creatinine levels for the diagnosis of acute rejection.

## Challenges And Future Directions

Different non-invasive biomarkers for the diagnosis and early prediction of acute rejection after kidney transplantation are under investigation. The markers discussed in this review are generally increased in their level during acute rejection, but crucial problems are caused by chronic processes. Whereas, increased CXCL9/10 levels is induced by IFNγ and seems largely related to acute forms of damage, elevated dd-cfDNA represents a general damage marker that may also typify chronic transplant damage. Despite the wealth of biomarker research in recent years, none of the markers have been permanently implemented in today's clinical practice as universally accepted diagnostic tool. This may be due to the fact that many studies in the past had a retrospective character and/or were performed in one center. Validation of initial results in an independent cohort has often been lacking. On top of that, sensitivity and specificity of the particular biomarker might have been limited.

Indeed, none of the biomarkers discussed have reached 100% for both sensitivity and specificity. To reach higher overall predictive value, a more comprehensive approach could be to combine different assays, whereby potential biomarkers assessed in material obtained from different parts of the body are integrated. As discussed, serum creatinine assessment has limited specificity. This means that changes in the levels may not only occur due to acute rejection but also to acute tubular necrosis, viral infection, and drug toxicity. Likewise, the biomarkers discussed in this review are hampered in their ability to distinguish acute rejection from other causes of acute transplant function decline, such as viral infection.

One of the main questions is whether and when clinical consequences to biomarker information can be made. Related to this is the question how many days in advance a particular analyte predicts the first clinical signs of rejection. Changes in blood dd-cfDNA levels (43, 44) and mRNA levels in urine and blood (28, 29) were detected at least 2 weeks prior to the onset of the rejection. These findings are clinically highly relevant, since early detection offers a broad window for therapeutic intervention.

Transcriptomics and dd-cfDNA assessment have been applied in non-invasive material in the context of kidney transplantation. Whereas, blood mRNA profiles offer a fairly high probability score for acute rejection, indeterminate risk calls (13–15% of cases) were frequently observed (29). Therefore, further evaluation of kSORT as an objective measure for acute rejection risk is ongoing, and randomized clinical trials are being performed to evaluate a kSORT-based monitoring strategy in the clinical follow-up of renal transplant recipients compared to standard monitoring protocols. This strategy should enable to evaluate if interventions based on peripheral blood signatures can positively influence the patient's outcome.

So far, usefulness of dd-cfDNA% as biomarker of acute kidney transplant rejection has not often been studied. One of the reasons may be the limited amount of cfDNA in the patient's blood plasma (10–15 ng/mL), the relatively low donor-derived fraction (1–5% of total cfDNA during rejection), and the high rate of genomic DNA contamination after prolonged incubation of conventional blood tubes (45). These limitations may be overcome by freshly obtaining higher volumes of blood plasma (5–10 mL) in specialized cfDNA-preserving blood tubes (45, 46), as quickly as possibly at the bedside. Such approach may be held back by the fact that high sample volumes of clinical material are not always available, and specialized blood tubes and extraction procedures are costly. Even when these hurdles are taken, there is a need for prospective, longitudinal studies investigating the role of dd-cfDNA as an early marker of rejection and other types of graft injury after kidney transplantation.

The question is whether and in which cases a biopsy may be abandoned. At time of acutely elevated serum creatinine, the biopsy remains the golden standard in distinguishing acute rejection from other causes of graft function decline. Furthermore, it allows to diagnose TCMR or ABMR. This has important implications for treatment, which is different between these conditions. The current biomarkers do not represent a satisfying substitute of conventional parameters in distinguishing TCMR and ABMR from each other and from other causes of transplant dysfunction. Since especially NPV of the biomarkers discussed is relatively high, they may complement serum creatinine measurement in predicting immune quiescence in the transplant and in deciding not to take a biopsy. Secondly, biomarkers may be superior to serum creatinine measurement in detecting the presence or absence of subclinical rejection, which normally would stay below the radar of detection when using merely serum creatinine as indicator. Subclinical rejection has an incidence of 5% within the first 6 months after transplantation (47) and significantly impacts long-term graft survival (48).

Whether clinical outcomes are positively affected by blood- and urine parameter driven interventions, in the absence of biopsies, remains to be determined by prospective randomized multicenter studies (26). The clinical utility of the biomarkers discussed needs confirmation in randomized control trials, thereby investigating a possible influence of molecular monitoring strategies, compared to standard monitoring protocols, on the patient's outcome.

## Author Contributions

ME wrote the majority of the article. EG and JY contributed parts of the text. KL, DA, and FC commented on the text.

### Conflict of Interest Statement

The authors declare that the research was conducted in the absence of any commercial or financial relationships that could be construed as a potential conflict of interest.
